# Complex lung segmentectomy: comparative perioperative outcomes of robotic and video-assisted approaches

**DOI:** 10.1007/s11748-026-02258-y

**Published:** 2026-01-31

**Authors:** Daisuke Nakamura, Daisuke Hara, Shuji Mishima, Yukihiro Terada, Hirotaka Kumeda, Kentaro Miura, Takashi Eguchi, Kazutoshi Hamanaka, Kimihiro Shimizu

**Affiliations:** 1 Division of General Thoracic Surgery, Department of Surgery, Shinshu University School of Medicine, 3-1-1 Asahi, Matsumoto, Japan; 2 Department of Cardiovascular and Thoracic Surgery, The University of Texas Southwestern Medical Center, Dallas, TX USA

**Keywords:** Robotic-assisted thoracic surgery, Video-assisted thoracoscopic surgery, Lung segmentectomy, Complex segmentectomy, Perioperative outcomes

## Abstract

**Objective:**

Segmentectomy is the standard treatment for small peripheral non-small cell lung cancer. While video-assisted thoracoscopic surgery (VATS) and robot-assisted thoracic surgery (RATS) are widely used, evidence focusing on complex segmentectomy (CS) is limited. Therefore, we aimed to compare the perioperative outcomes of VATS and RATS for segmentectomy, focusing on CS.

**Methods:**

We retrospectively reviewed 497 segmentectomies performed at Shinshu University Hospital (January 2020 and February 2025). After excluding cases of thoracotomy, benign tumors, and prior lung resection, 391 patients (268 CS) were included. Propensity score matching (PSM) generated 159 VATS–RATS pairs for all segmentectomies and 99 pairs for CS. The perioperative outcomes and complications were compared.

**Results:**

For all segmentectomies, the operative time was longer with RATS (median, 219 vs. 192 min; *p* < 0.001), whereas blood loss, drainage duration, length of stay, reoperation, and readmission were comparable. Although not significantly different, arrhythmia incidence was less frequent in the RATS group after PSM (1.9% vs. 5.7%; *p* = 0.077). In CS, RATS had longer operative time (220 vs. 189 min; *p* < 0.001) but fewer Clavien–Dindo grade ≥ 2 complications (10.1% vs. 20.2%; *p* = 0.047) and arrhythmia incidences (1.0% vs. 7.1%; *p* = 0.030). No 90-day mortalities were observed.

**Conclusions:**

Despite longer operative times, RATS achieved perioperative outcomes comparable to VATS and was associated with a lower incidence of postoperative arrhythmia, particularly in CS, potentially related to a reduced extent of lung resection.

**Supplementary Information:**

The online version contains supplementary material available at 10.1007/s11748-026-02258-y.

## Introduction

Large randomized prospective studies have established segmentectomy as the standard of care for small peripheral non-small cell lung cancer [1, 2]. Minimally invasive approaches, such as video-assisted thoracoscopic surgery (VATS) and robot-assisted thoracic surgery (RATS), have become standard [3, 4]. Complex segmentectomy (CS) entails greater anatomical variability and technical difficulty than simple procedures [5]; however, comparative evidence, specifically for CS, remains scarce. Therefore, we aimed to compare the perioperative outcomes of VATS and RATS for all segmentectomies at our institution, with a focus on CS, using propensity score matching (PSM) to mitigate baseline imbalances. We also aimed to assess the perioperative outcomes and postoperative complications.

## Methods

### Study cohort

**Fig. 1:**
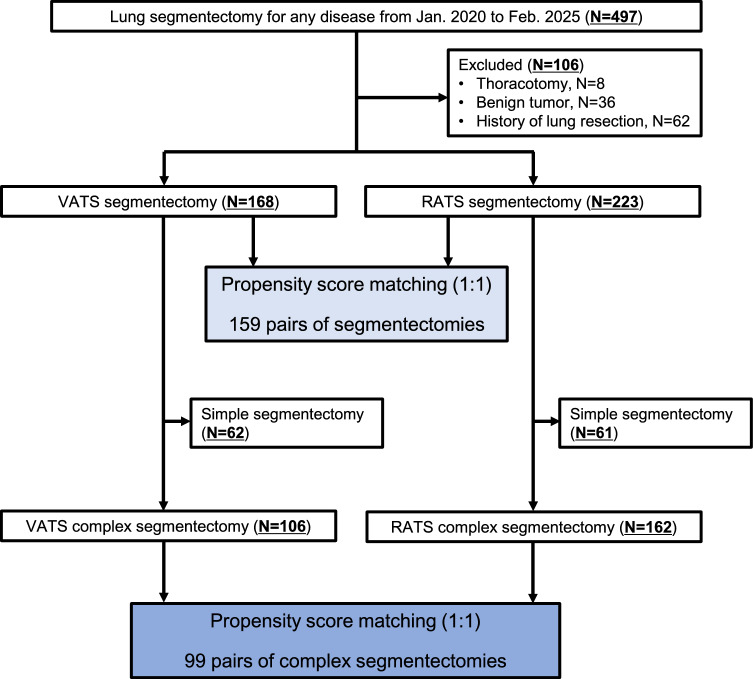
Patient flowchart. *RATS* Robotic-assisted thoracic surgery, *VATS* Video-assisted thoracoscopic surgery

This retrospective study was approved by the Institutional Review Board of Shinshu University Hospital (Project ID 6609). An opt-out approach was employed, rather than obtaining written informed consent from each patient. The study adhered to the principles outlined in the Declaration of Helsinki. We retrospectively collected data of 497 segmentectomies performed at Shinshu University Hospital between January 2020 and February 2025. After excluding cases of thoracotomy (n = 8), benign tumors (n = 36), and prior lung resection (n = 62), 391 patients were included, 268 of whom underwent CS (Fig. 1).

### Indication of segmentectomy

Segmentectomy was indicated for biopsy-proven or suspected primary lung cancers with a total tumor size ≤ 2 cm, ground-glass–dominant nodules > 2 cm, or cases considered high-risk for lobectomy because of reduced respiratory function, significant comorbidities, advanced age, poor performance status. Patients with metastatic and benign lung tumors who were ineligible for wedge resection were candidates for segmentectomy. The borderline and indeterminate cases were discussed at a multidisciplinary conference.

### Definition of simple and complex segmentectomy

Simple segmentectomy included S^6^, basal, left upper division, and lingular segmentectomies. Complex segmentectomy was defined as a procedure other than simple segmentectomy [5].

### Surgical procedures

Preoperative computed tomography (CT) was performed for all patients, and the REVORAS (Ziosoft, Tokyo, Japan) software generated detailed three-dimensional CT (3D-CT) visualizations of the pulmonary vessels and bronchi. The 3D-CT imaging facilitates the precise planning of each segmentectomy [6]. VATS was performed entirely under thoracoscopic visualization using four ports. The surgeon performed the procedure through one port and a 2–3 cm utility incision, whereas the assistant used a 2–3 cm incision (Video 1). The RATS was performed under CO₂ insufflation at a pressure of 5–8 mm Hg using the da Vinci Si and Xi Surgical Systems (Intuitive Surgical, Sunnyvale, CA, USA). Our standard robotic instrumentation included a 0° camera, Maryland bipolar forceps in the right arm, fenestrated bipolar forceps in the left arm, and Cadiere or tip-up forceps for retraction, along with a vessel sealer or SureForm stapler extension through any port. Port placement followed a modified Cerfolio-style configuration [7, 8]. In most cases, the intersegmental plane was determined by intravenous injection of indocyanine green. In some cases, the inflation–deflation method was used for delineation. The intersegmental plane was primarily divided using a stapler; however, electrocautery or other techniques were used when necessary (Video 2).

### Statistical analysis

All statistical analyses were performed using the IBM SPSS Statistics version 30 software (IBM, Armonk, NY, USA). Data are presented as numbers (percentages) or medians (interquartile ranges). In cases of extended segmentectomy or additional wedge resection, we added 0.5 to the count. The association between variables was assessed using the Mann–Whitney U test for continuous variables and Fisher’s exact test or chi-squared test for categorical variables. To eliminate imbalances in baseline characteristics between the VATS and RATS groups, PSM was performed using the following covariates: age, sex, body mass index, performance status, smoking history, chronic obstructive pulmonary disease, interstitial pneumonia, diabetes mellitus, ischemic heart disease, cerebrovascular disease, percentage predicted forced vital capacity, percentage predicted forced expiratory volume in 1s, percentage predicted diffusing capacity of the lung for carbon monoxide, and type of segmentectomy. A 1:1 nearest-neighbor matching was conducted with a caliper width of 0.05, resulting in 159 matched patients for all segmentectomies and 99 matched patients for CS. Statistical significance was set at *p* <  0.05.

The primary endpoint was the incidence of postoperative complications, defined as Clavien–Dindo (CD) grade ≥ 2, within 30 days after surgery.

## Results

### Distribution of procedures by surgical approach

Supplemental Fig. S1 shows the distribution of segmentectomy procedures by surgical approach. Simple segmentectomy was significantly more prevalent in the VATS group (Table 1, *p* = 0.044), and approximately 27% of CSs involved either combined or single subsegmentectomy.

**Table 1:** Patient characteristics in all segmentectomy cases

Characteristics	Before propensity score matching (n = 391)			After propensity score matching (n = 318)		
	VATS (n = 168)	RATS (n = 223)	*p* value	VATS (n = 159)	RATS (n = 159)	*p* value
Age (years)	72 (64–76)	72 (65–76)	0.592	72 (64–76)	71 (66–76)	0.728
Gender			0.757			0.259
Male	87 (52)	119 (53)		83 (52)	93 (59)	
Female	81 (48)	104 (47)		76 (48)	66 (41)	
BMI (kg/m^2^)	23 (21–25)	23 (21–25)	0.985	23 (21–25)	22 (21–25)	0.691
Performance status			0.685			0.605
0/1/2	152 (91) /16 (9) / 0 (0)	201 (90) /21 (9) / 1 (1)		143 (90) /16 (10) / 0 (0)	142 (89) /16 (10) / 1 (1)	
Smoking			0.323			0.500
Yes	88 (52)	128 (57)		82 (52)	88 (55)	
No	80 (48)	95 (43)		77 (48)	71 (45)	
Brinkman index			0.691			0.895
> = 800	40 (24)	57 (26)		37 (23)	38 (24)	
< 800	128 (76)	166 (74)		122 (77)	121 (76)	
COPD			0.720			0.665
Yes	30 (18)	43 (19)		28 (18)	31 (19)	
No	138 (82)	180 (81)		131 (82)	128 (81)	
Interstitial pneumonia			0.114			0.777
Yes	12 (7)	8 (4)		7 (4)	6 (4)	
No	156 (93)	215 (96)		152 (96)	153 (96)	
Diabetes mellitus			0.769			0.882
Yes	29 (17)	36 (16)		27 (17)	28 (18)	
No	139 (83)	187 (84)		132 (83)	131 (82)	
Ischemic cardiac disease			0.118			0.332
Yes	7 (4)	18 (8)		7 (4)	11 (7)	
No	161 (96)	205 (92)		152 (96)	148 (93)	
Cerebrovascular disease			**0.008**			0.702
Yes	5 (3)	22 (10)		4 (2)	3 (2)	
No	163 (97)	201 (90)		155 (98)	156 (98)	
History of arrhythmia			0.057			0.145
Yes	9 (5)	24 (11)		9 (6)	16 (10)	
No	159 (95)	199 (89)		150 (94)	143 (90)	
Antiarrhythmic medications			0.268			0.520
Yes	4 (2)	10 (4)		4 (3)	6 (4)	
No	164 (98)	213 (96)		155 (97)	153 (96)	
%FVC (%)	106 (96–117)	108 (96–119)	0.650	107 (96–117)	107 (94–118)	0.815
FEV1.0/FVC (%)	77 (71–83)	77 (70–82)	0.943	77 (71–83)	77 (70–82)	0.594
%FEV1.0 (%)	101 (89–115)	102 (89–114)	0.833	101 (89–115)	100 (88–111)	0.443
%DLco (%)	87 (72–99)	88 (72–103)	0.204	87 (72–100)	87 (72–104)	0.480
Tumor size (cm)	1.5 (1.2–2.2)	1.6 (1.2–2.2)	0.647	1.5 (1.2–2.1)	1.6 (1.1–2.2)	0.730
Type of tumor			0.154			0.332
Primary lung cancer	133 (79)	191 (86)		125 (79)	134 (84)	
Metastatic tumor	32 (19)	31 (14)		31 (19)	24 (15)	
Other	3 (2)	1 (0.4)		3 (2)	1 (1)	
Type of segmentectomy			**0.044**			0.723
Simple	62 (37)	61 (27)		56 (35)	53 (33)	
Complex	106 (63)	162 (73)		103 (65)	106 (67)	

Data are shown as number (%) or median (25–75 percentiles). BMI, body mass index; COPD, chronic obstructive pulmonary disease; %DLco, percent predicted diffusing capacity of the lung for carbon monoxide; %FEV1.0, percent predicted forced expiratory volume in 1s; %FVC, percent predicted forced vital capacity; RATS, robotic-assisted thoracic surgery; VATS, video-assisted thoracoscopic surgery, statistical significance was set at p < 0.05.

### Patient characteristics

Table 1 shows patient characteristics before and after PSM. In total, 391 patients were included in this study, of whom 168 underwent VATS and 223 underwent RATS. Before PSM, cerebrovascular disease was significantly more prevalent in the RATS group (*p* = 0.008). After PSM, 159 patients were matched in each group, and no significant differences were observed in baseline patient characteristics between groups. After PSM, the prevalence of preoperative arrhythmia history and the use of antiarrhythmic medications were comparable between the two groups.

### Perioperative outcomes

Table 2 shows the perioperative outcomes before and after PSM. The operative time was significantly longer in the RATS group (219 [194–259] vs. 192 [167–225] min, *p* <  0.001), and the median console time of RATS was 159 min after PSM. Although not significant, a longer postoperative drainage duration was observed in the RATS group (2.0 [2.0–3.0] vs. 2.0 [2.0–2.5] days, *p* = 0.070) after PSM. No significant differences were observed between the two groups in terms of blood loss, length of postoperative hospital stay, reoperation, or readmission after PSM. None of the patients in either group required a conversion to thoracotomy.

**Table 2:** perioperative outcomes in all segmentectomy cases

Variables	Before propensity score matching (n=391)			After propensity score matching (n=318)		
	VATS (n=168)	RATS (n=223)	*p* value	VATS (n=159)	RATS (n=159)	*p* value
Number of resected subsegments	3 (2–4)	3 (2–4)	0.403	3 (2–4)	3 (2–4)	0.527
Additional wedge resection			0.569			1.000
Yes	11 (7)	18 (8)		10 (6)	10 (6)	
No	157 (93)	205 (92)		149 (94)	149 (94)	
Subsegmentectomy			0.133			0.442
Yes	39 (23)	67 (30)		38 (24)	44 (28)	
No	129 (77)	156 (70)		121 (76)	115 (72)	
Mediastinal lymph node dissection			0.174			0.403
Yes	19 (11)	36 (16)		18 (11)	23 (14)	
No	149 (89)	187 (84)		141 (89)	136 (86)	
Console time (min)	**–**	164 (141−196)	**–**	**–**	159 (135–196)	**-**
Operation time (min)	192 (165–226)	225 (195–262)	**<.001**	192 (167–225)	219 (194–259)	**<.001**
Blood loss (ml)	20 (10-50)	10 (10-30)	0.058	20 (10–50)	10 (10-38)	0.306
Conversion to thoracotomy	0 (0)	0 (0)	**–**	0 (0)	0 (0)	**–**
Length of drainage (days)	2 (2–3)	2 (2–3)	0.416	2 (2.0–2.5)	2 (2.0-3.0)	0.070
Length of hospital stays (days)	6 (5–8)	6 (5–8)	0.077	6 (5-8)	6 (5–8)	0.364
Reoperation	2 (1.2)	3 (1.3)	0.893	2 (1.3)	1 (0.6)	0.562
Readmission	3 (1.8)	5 (2.2)	0.752	2 (1.3)	4 (2.5)	0.410
Postoperative complications, CD ≧ Grade 2	34 (20.2)	30 (13.5)	0.073	31 (19.5)	23 (14.5)	0.232
Postoperative hemorrhage	0 (0)	2 (0.9)	0.218	0 (0)	1 (0.6)	0.317
Prolonged air leak	7 (4.2)	12 (5.4)	0.580	7 (4.4)	8 (5.0)	0.791
Late onset air leak	0 (0)	1 (0.4)	0.385	0 (0)	1 (0.6)	0.317
Arrhythmia	11 (6.5)	5 (2.2)	**0.033**	9 (5.7)	3 (1.9)	0.077
Acute myocardial infarction	0 (0)	1 (0.4)	0.385	0 (0)	1 (0.6)	0.317
Pneumonia	6 (3.6)	7 (3.1)	0.813	5 (3.1)	6 (3.8)	0.759
Empyema	2 (1.2)	0 (0)	0.102	2 (1.3)	0 (0)	0.156
Atelectasis	1 (0.6)	2 (0.9)	0.735	1 (0.6)	2 (1.3)	0.562
Respiratory failure	2 (1.2)	2 (0.9)	0.775	1 (0.6)	2 (1.3)	0.562
Acute exacerbation of interstitial pneumonia	0 (0)	2 (0.9)	0.218	0 (0)	1 (0.6)	0.317
Acute respiratory distress syndrome	1 (0.6)	0 (0)	0.249	1 (0.6)	0 (0)	0.317
Bronchopleural fistula	2 (1.2)	0 (0)	0.102	2 (1.3)	0 (0)	0.156
Prolonged inflammatory response	1 (0.6)	2 (0.9)	0.735	1 (0.6)	2 (1.3)	0.562
Pulmonary embolism / deep venous thrombosis	5 (3.0)	2 (0.9)	0.125	5 (3.1)	2 (1.3)	0.252
Delirium	0 (0)	2 (0.9)	0.218	0 (0)	1 (0.6)	0.317
Others	2 (1.2)	2 (0.9)	0.775	2 (1.3)	1 (0.6)	0.562
Postoperative respiratory complications	19 (11.3)	22 (9.9)	0.645	17 (10.7)	18 (11.3)	0.858
Postoperative complications, CD ≧ Grade 3	14 (8.3)	14 (6.3)	0.435	14 (8.8)	9 (5.7)	0.279
90-days mortality	0 (0)	0 (0)	**-**	0 (0)	0 (0)	**-**

Data are shown as number (%) or median (25–75 percentiles). CD, Clavien-Dindo; RATS, robotic-assisted thoracic surgery; VATS, video-assisted thoracoscopic surgery, statistical significance was set at p < 0.05.

Regarding postoperative complications within 30 days post-surgery, postoperative arrhythmias were less frequent in the RATS group (*p* = 0.077) after PSM; however, no significant difference was observed in postoperative complications of CD grade ≥ 2. Similarly, no significant differences were observed regarding postoperative respiratory complications such as prolonged air leak or pneumonia, as well as severe complications of CD grade ≥ 3. None of the patients died within 90 days post-surgery.

### Complex segmentectomy

CS was performed in 268 (69%) patients. Among them, 106 underwent VATS and 162 underwent RATS. Table 3 shows the CS patient characteristics before and after PSM. Before PSM, interstitial pneumonia was more frequent in the VATS group, whereas cerebrovascular disease was significantly more common in the RATS group (*p* = 0.007). After PSM, 99 patients were matched in each group, and no significant differences were observed in the baseline patient characteristics between groups. After PSM, preoperative arrhythmia history and antiarrhythmic medication use were also well balanced between the two groups.

**Table 3:** Patient characteristics in complex segmentectomy cases

Characteristics	Before propensity score matching (n = 268)			After propensity score matching (n = 198)		
	VATS (n = 106)	RATS (n = 162)	*p* value	VATS (n = 99)	RATS (n = 99)	*p* value
Age (years)	71 (63–75)	72 (64–76)	0.460	71 (64–75)	70 (63–76)	0.773
Gender			0.389			0.569
Male	46 (43)	79 (49)		45 (46)	49 (49)	
Female	60 (57)	83 (51)		54 (54)	50 (51)	
BMI (kg/m^2^)	23 (21–25)	22 (21–25)	0.634	22 (21–25)	22 (21–24)	0.403
Performance status			0.705			0.605
0 / 1 / 2	96 (91) / 10 (9) / 0 (0)	147 (90) / 14 (9) / 1 (1)		90 (91) / 9 (9) / 0 (0)	89 (90) / 9 (9) / 1 (1)	
Smoking			0.399			1.000
Yes	52 (49)	88 (54)		50 (51)	50 (51)	
No	54 (51)	74 (46)		49 (49)	49 (49)	
Brinkman index			0.621			1.000
> = 800	19 (18)	33 (20)		17 (17)	16 (16)	
< 800	87 (82)	129 (80)		82 (83)	83 (84)	
COPD			0.892			1.000
Yes	17 (16)	27 (17)		17 (17)	17 (17)	
No	89 (84)	135 (83)		82 (83)	82 (83)	
Interstitial pneumonia			0.091			0.651
Yes	6 (6)	3 (2)		3 (3)	2 (2)	
No	100 (94)	159 (98)		96 (97)	97 (98)	
Diabetes mellitus			0.950			0.692
Yes	16 (15)	24 (15)		14 (14)	16 (16)	
No	90 (85)	138 (85)		85 (86)	83 (84)	
Ischemic cardiac disease			0.665			1.000
Yes	7 (7)	13 (8)		7 (7)	7 (7)	
No	99 (93)	149 (92)		92 (93)	92 (93)	
Cerebrovascular Disease			**0.007**			0.561
Yes	2 (2)	17 (10)		2 (2)	1 (1)	
No	104 (98)	145 (90)		97 (98)	98 (99)	
History of arrhythmia			0.127			0.297
Yes	7 (7)	20 (12)		6 (6)	10 (10)	
No	99 (93)	142 (88)		93 (94)	89 (90)	
Antiarrhythmic medications			0.388			1.000
Yes	4 (4)	10 (6)		4 (4)	4 (4)	
No	102 (96)	152 (94)		95 (96)	95 (96)	
%FVC (%)	109 (99–118)	110 (96–120)	0.860	109 (99–117)	112 (97–122)	0.623
FEV1.0/FVC (%)	78 (71–83)	77 (70–82)	0.471	77 (71–83)	77 (70–82)	0.652
%FEV1.0 (%)	105 (93–117)	105 (88–116)	0.433	104 (93–116)	105 (90–114)	0.650
%DLco (%)	87 (73–100)	89 (75–103)	0.296	88 (75–100)	89 (73–102)	0.972
Tumor size (cm)	1.5 (1.1–2.0)	1.6 (1.1–2.0)	0.604	1.5 (1.1–2.0)	1.6 (1.1–2.1)	0.808
Type of tumor			0.105			0.187
Primary lung cancer	82 (77)	137 (85)		75 (76)	83 (84)	
Metastatic tumor	22 (21)	25 (15)		22 (22)	16 (16)	
Other	2 (2)	0 (0)		2 (2)	0 (0)	

Data are shown as number (%) or median (25–75 percentiles). BMI, body mass index; COPD, chronic obstructive pulmonary disease; %DLco, percent predicted diffusing capacity of the lung for carbon monoxide; %FEV1.0, percent predicted forced expiratory volume in 1 s; %FVC, percent predicted forced vital capacity; RATS, robotic-assisted thoracic surgery; VATS, video-assisted thoracoscopic surgery, statistical significance was set at p < 0.05.

Table 4 shows the perioperative outcomes before and after PSM in the patients who underwent CS. The operative time was significantly longer in the RATS group (220 [193–256] vs. 189 [167–213] min, *p* <  0.001) than in the VATS group, and the median console time was 159 min after PSM. No significant differences were observed between the two groups in terms of blood loss, postoperative drainage duration, length of postoperative hospital stay, reoperation, or readmission after PSM. The proportion of patients who underwent mediastinal lymph node dissection was higher in the RATS group.

**Table 4:** Perioperative outcomes in complex segmentectomy cases

Variables	Before propensity score matching (n = 268)			After propensity score matching (n = 198)		
	VATS (n = 106)	RATS (n = 162)	*p* value	VATS (n = 99)	RATS (n = 99)	*p* value
Number of resected subsegments	2.3 (2–3.5)	2.5 (2–3)	0.964	2.5 (2.0–4.0)	2.0 (2.0–3.0)	0.175
Additional wedge resection			0.439			0.788
Yes	7 (7)	15 (9)		7 (7)	8 (8)	
No	99 (93)	147 (91)		92 (93)	91 (92)	
Subsegmentectomy			0.455			0.466
Yes	39 (37)	67 (41)		36 (36)	41 (41)	
No	67 (63)	95 (59)		63 (64)	58 (59)	
Mediastinal lymph node dissection			0.054			0.070
Yes	7 (7)	23 (14)		7 (7)	15 (15)	
No	99 (93)	139 (86)		92 (93)	84 (85)	
Console time (min)	**–**	168 (141–200)	**–**	**–**	159 (129–195)	**–**
Operation time (min)	186 (164–211)	230 (198–265)	** < .001**	189 (167–213)	220 (193–256)	** < .001**
Blood loss (ml)	20 (10–30)	10 (10–40)	0.451	20 (10–30)	10 (10–50)	0.810
Conversion to thoracotomy	0 (0)	0 (0)	**–**	0 (0)	0 (0)	**–**
Length of drainage (days)	2 (2–2)	2 (2–3)	0.304	2 (2–2)	2 (2–2)	0.111
Length of hospital stays (days)	6 (5–8)	6 (5–7)	0.082	6 (5–8)	6 (5–7)	0.204
Reoperation	0 (0)	1 (0.6)	0.418	0 (0)	1 (1)	0.316
Readmission	0 (0)	3 (1.9)	0.159	0 (0)	1 (1)	0.316
Postoperative complications, CD ≧ Grade 2	22 (20.8)	16 (9.9)	**0.013**	20 (20.2)	10 (10.1)	**0.047**
Postoperative hemorrhage	0 (0)	1 (0.6)	0.418	0 (0)	1 (1)	0.316
Prolonged air leak	3 (2.8)	7 (4.3)	0.529	2 (2.0)	3 (3.0)	0.651
Late onset air leak	0 (0)	1 (0.6)	0.418	0 (0)	0 (0)	**–**
Arrhythmia	8 (7.5)	3 (1.9)	**0.022**	7 (7.1)	1 (1.0)	**0.030**
Acute myocardial infarction	0 (0)	1 (0.6)	0.418	0 (0)	1 (1.0)	0.316
Pneumonia	5 (4.7)	2 (1.2)	0.081	5 (5.1)	2 (2.0)	0.248
Empyema	1 (0.9)	0 (0)	0.216	1 (1.0)	0 (0)	0.316
Atelectasis	1 (0.9)	1 (0.6)	0.762	1 (1.0)	1 (1.0)	1.000
Respiratory failure	1 (0.9)	1 (0.6)	0.762	1 (1.0)	1 (1.0)	1.000
Acute exacerbation of interstitial pneumonia	0 (0)	0 (0)	**–**	0 (0)	0 (0)	**–**
ARDS	1 (0.9)	0 (0)	0.216	1 (1.0)	0 (0)	0.316
Bronchopleural fistula	1 (0.9)	0 (0)	0.216	1 (1.0)	0 (0)	0.316
Prolonged inflammatory response	0 (0)	2 (1.2)	0.251	0 (0)	2 (2.0)	0.155
Pulmonary embolism / deep venous thrombosis	3 (2.8)	0 (0)	**0.031**	3 (3.0)	0 (0)	0.081
Delirium	0 (0)	1 (0.6)	0.418	0 (0)	1 (1.0)	0.316
Others	1 (0.9)	1 (0.6)	0.762	1 (1.0)	0 (0)	0.316
Postoperative respiratory complications, overall	12 (11.3)	11 (6.8)	0.195	11 (11.1)	6 (6.1)	0.205
Postoperative complications, CD ≧ Grade 3	9 (8.5)	8 (4.9)	0.243	8 (8.6)	3 (3.2)	0.389
90-days mortality	0 (0)	0 (0)	**–**	0 (0)	0 (0)	**–**

Data are shown as number (%) or median (25–75 percentiles). CD, Clavien-Dindo; RATS, robotic-assisted thoracic surgery; VATS, video-assisted thoracoscopic surgery, statistical significance was set at p < 0.05.

After PSM, postoperative complications of CD grade ≥ 2 were significantly less frequent in the RATS group (*p* = 0.047). While postoperative arrhythmias were significantly less common in the RATS group (*p* = 0.030), no significant differences were observed in respiratory complications or severe complications of CD grade ≥ 3.

## Discussion

This retrospective study compared short-term outcomes of VATS and RATS in patients undergoing segmentectomy. Although several large-scale studies have evaluated these approaches, a notable strength of this study lies in its focused analysis of CS and the use of PSM to minimize patient background bias.

Previous reports have shown that minimally invasive approaches, including VATS and RATS, result in fewer complications and shorter hospital stays than open thoracotomy [9, 10]. However, studies comparing RATS and VATS in lung segmentectomy generally reported similar perioperative outcomes, with RATS often incurring higher costs [11, 12]. Our study also demonstrated comparable perioperative outcomes between the two approaches after PSM, including blood loss, length of hospital stay, reoperation, and readmission rates. However, operative time was significantly longer in the RATS group. Notably, console time during RATS was approximately 30 min shorter than the total VATS time, suggesting that the longer RATS duration was primarily owing to robot setup rather than the surgical procedure itself. The extended preparation time likely reflects our university hospital setting, where trainees participate in robotic setup. With growing experience and improved robotic systems, setup and console times expected to decrease.

Atrial fibrillation is the most common type of postoperative arrhythmia, with a reported incidence of 9–19% [13, 14]. In this study, 13 of the 16 patients with arrhythmia had atrial fibrillation. In our cohort, the incidence of postoperative arrhythmias tended to be lower in the RATS group, and was significantly lower in the group undergoing CS. The advantages of RATS include a 3D, high-magnification view and articulated instruments that enable precise manipulation, making it particularly well-suited for lung segmentectomy, which requires meticulous vascular handling and dissection. Therefore, compared to VATS, RATS may facilitate more precise and limited resections, including subsegmentectomy, which can be advantageous for CS. Supplemental Tables S1 and S2 present the series of segmentectomies performed in this study cohort, demonstrating their high complexity. Among the patients who underwent CS after PSM, the distribution of subsegmentectomy was comparable between the two groups; however, the median number of resected subsegments was slightly lower in the RATS group (VATS 2.5 vs. RATS 2.0, Table 4). We previously reported that limiting the number of subsegmental resections may reduce the incidence of postoperative atrial fibrillation, which could decrease the rate of other non-cancer-related events [15]. Motono et al. reported that lobectomy or greater resection was a significant risk factor for postoperative arrhythmia [16]. Accordingly, the lower incidence of postoperative arrhythmias observed after RATS-CS may, at least in part, reflect differences in the extent of parenchymal resection. This interpretation is supported by the comparable prevalence of a preoperative history of arrhythmias and the use of antiarrhythmic medications between the two groups after PSM (Table 3). Because postoperative respiratory or cardiac function was not assessed in this study, the mechanism underlying the association between surgical approach and postoperative arrhythmias requires further investigation.

No significant differences were observed between the two groups in terms of respiratory complications or severe complications classified as CD grade ≥ 3. In a multicenter study, Zhang et al. reported no significant differences in the incidence of postoperative pneumonia, prolonged air leaks, or major complications between VATS and RATS segmentectomy [11]. Because respiratory complications are mainly influenced by underlying lung conditions such as emphysema [17], no difference was observed between the two minimally invasive approaches.

This study had certain limitations. This was a retrospective study conducted at a single institution. In addition, temporal factors and learning-curve effects (including surgeon experience with each approach) could have influenced the outcomes and were not fully accounted for. Additionally, postoperative respiratory function and quality of life, as well as oncological outcomes such as recurrence and long-term prognosis, were not evaluated. Although RATS is associated with higher costs than VATS, we did not conduct a cost-effectiveness analysis. Future studies should examine postoperative respiratory function, quality of life, and cost-effectiveness to further validate the benefits of RATS in CS.

## Conclusion

Although RATS was associated with a significantly longer operative time compared with VATS, other perioperative outcomes were comparable between the two groups. Postoperative arrhythmia occurred less frequently after RATS, particularly in CS, and this association may be influenced by the reduced extent of lung resection.

## Supplementary Information

Below is the link to the electronic supplementary material.

Supplementary file 1 (PDF 64 KB)

Supplementary file 2 (PDF 190 KB)

Supplementary file 3 (MPG 408536 KB)

Supplementary file 4 (MPG 368142 KB)

## Declarations

### Data availability

The data underlying this article cannot be shared publicly due to for the privacy of individuals that participated in the study. The data will be shared on reasonable request to the corresponding author.
