# Supplementary material for: Complex lung segmentectomy: comparative perioperative outcomes of robotic and video-assisted approaches
Source: Gen Thorac Cardiovasc Surg. 2026 Jan 31;74(7):686–94. doi: 10.1007/s11748-026-02258-y (PMC13283155; doi:10.1007/s11748-026-02258-y)

Supplemental Figure S1. The pie chart show the distribution of surgical procedures in robotic and video-assisted surgery cases.

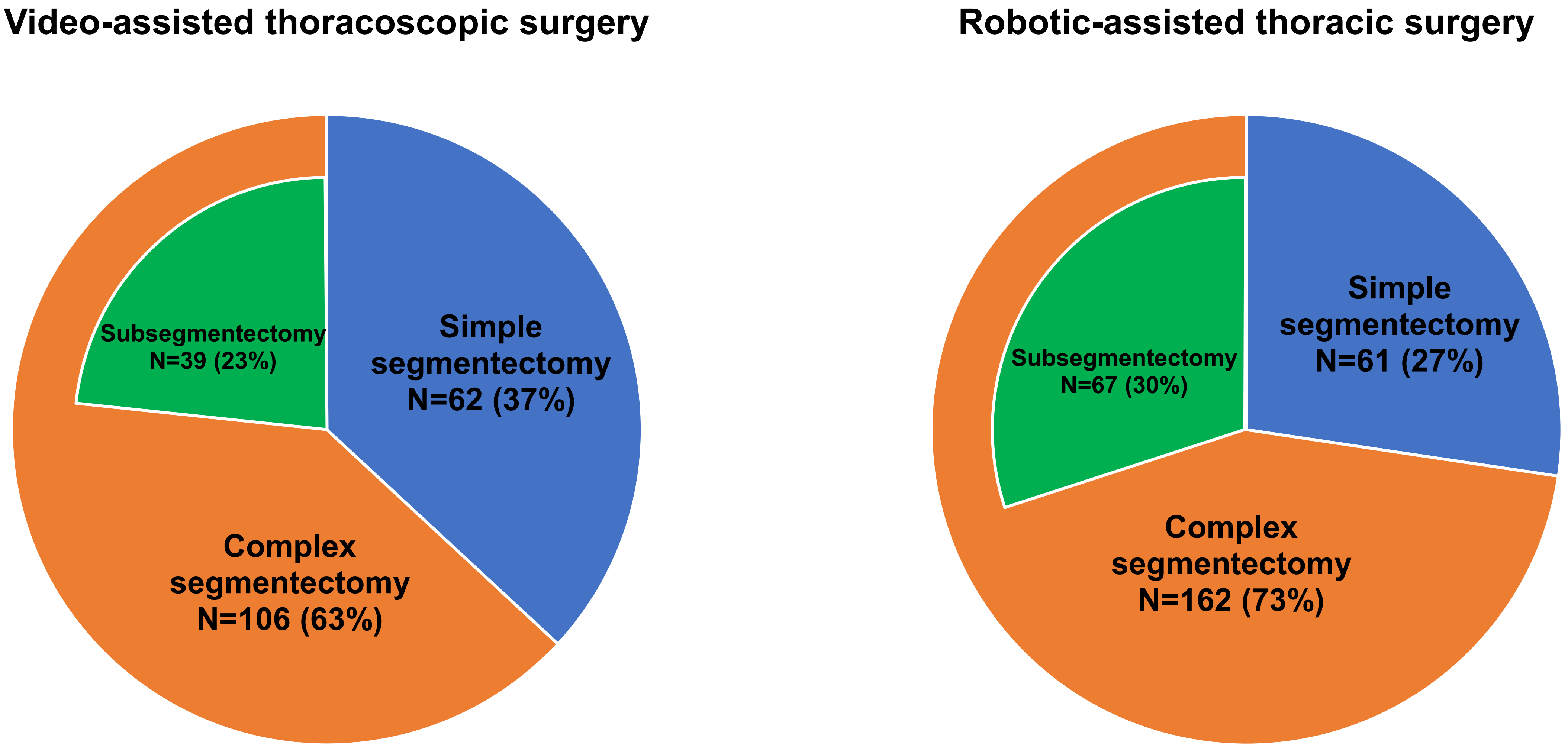

Supplement: Supplementary file 1 — (PDF 64 KB) [file 11748_2026_2258_MOESM1_ESM.pdf]
