# Supplementary material for: Complex lung segmentectomy: comparative perioperative outcomes of robotic and video-assisted approaches
Source: Gen Thorac Cardiovasc Surg. 2026 Jan 31;74(7):686–94. doi: 10.1007/s11748-026-02258-y (PMC13283155; doi:10.1007/s11748-026-02258-y)
Supplement: Supplementary file 2 — (PDF 190 KB) [file 11748_2026_2258_MOESM2_ESM.pdf]

Supplemental Table S1. Details of the surgical procedure and the number of cases in video-assisted thoracoscopic surgery cases.

Right side (n=75)				Left side (n=93)			
Simple segmentectomy (n=17)		Complex segmentectomy (n=58)		Simple segmentectomy (n=45)		Complex segmentectomy (n=48)	
S^{6}	14	S^{1}	5	Left upper division	14	S^{1+2}	9
Basal	3	$S^{1}a$	2	Lingula	8	$S^{1+2}a+b$	3
		$S^{1}b$	1	S^{6}	14	S1+2b	1
		$S^{1}+S^{2}$	1	Basal	9	$S^{1+2}c$	4
		$S^{1}+S^{2}a$	1			$S^{1+2}+S^{3}a$	1
		$S^{1}a+S^{2}$	2			$S^{1+2}+S^{3}c$	1
		$S^{1}b+S^{3}$	1			S^{3}	1
		$S^{1}b+S^{3}b$	1			$S^{3}b$	2
		S^{2}	7			$S^{3}b+c$	1
		$S^{2}b+S^{3}a$	3			$S^{3}+S^{4}+S^{5}a$	1
		S^{3}	11			$S^{3}a+b+S^{4}+S^{5}$	1
		$S^{3}b$	3			$S^{3}b+S^{4}$	1
		$S^{3}a+S^{4}a$	1			S^{5}	2
		S^{4}	3			$S^{5}+S^{8}$	1
		S^{5}	1			$S^{6}b$	1
		$S^{5}b$	1			$S^{6}+S^{*}$	1
		$S^{6}+S^{9}a$	1			$S^{6}+S^{10}$	1
		$S^{6}+S^{8}a+S^{9}a$	1			$S^{6}+S^{10}a$	1
		S^{7}	2			S^{8}	4
		$S^{7}+S^{10}$	1			$S^{8}b$	2
		S^{8}	1			$S^{8}+S^{9}$	3
		$S^{8}+S^{9}$	1			$S^{9}+S^{10}$	3
		$S^{8}+S^{9}+S^{10}$	2			$S^{9}a+S^{10}$	1
		$S^{9}+S^{10}$	3			S^{10}	2
		S^{10}	2				

Data are presented as resected segments, and number of patients.

Supplemental Table S2. Details of the surgical procedure and the number of cases in robotic -assisted thoracoscopic surgery cases.

Right side (n=112)				Left side (n=111)			
Simple segmentectomy (n=15)		Complex segmentectomy (n=97)		Simple segmentectomy (n=46)		Complex segmentectomy (n=65)	
S^{6}	11	S^{1}	13	Left upper division	16	S^{1+2}	8
Basal	4	$S^{1}a$	2	Lingula	12	$S^{1+2}a$	1
		$S^{1}b$	1	S^{6}	13	$S^{1+2}b$	1
		$S^{1}+S^{2}$	3	Basal	5	$S^{1+2}a+b$	2
		$S^{1}+S^{2}a$	1			$S^{1+2}b+c$	2
		$S^{1}+S^{2}ai$	1			$S^{1+2}+S^{6}$	1
		$S^{1}+S^{3}a$	1			$S^{1+2}+S^{3}a$	3
		$S^{1}+S^{3}ai$	1			$S^{1+2}a+S^{3}$	1
		$S^{1}+S^{4}$	1			$S^{1+2}a+S^{3}b+c$	1
		$S^{1}+S^{9}+S^{10}$	1			$S^{1+2}a+S^{3}c$	2
		$S^{1}a+S^{2}$	4			$S^{1+2}aii+S^{3}b+c$	1
		$S^{1}a+S^{2}a$	1			$S^{1+2}a+b+S^{3}c$	1
		$S^{1}b+S^{3}$	1			$S^{1+2}ci+S^{4}a$	1
		S^{2}	11			S^{3}	8
		$S^{2}b$	1			$S^{3}b$	2
		$S^{2}+S^{3}a$	2			$S^{3}c$	1
		$S^{2}+S^{6}$	1			$S^{3}b+c$	1
		$S^{2}b+S^{3}a$	3			$S^{3}a+bi+S^{4}$	1
		$S^{2}b+S^{5}$	1			$S^{3}b+S^{4}$	1
		S^{3}	11			$S^{3}b+c+S^{8}$	1
		$S^{3}b$	5			$S^{4}+S^{5}+S^{6}c+S^{9}+S^{10}$	1
		$S^{3}+S^{5}$	1			$S^{4}a+S^{5}$	1
		S^{4}	2			$S^{5}a$	1
		$S^{4}a$	1			$S^{6}+S^{*}$	1
		$S^{4}+S^{7}$	1			$S^{6}+S^{10}$	2
		S^{5}	3			S^{7}	1
		$S^{6}+S^{7}$	1			S^{8}	3
		$S^{6}+S^{*}$	1			$S^{8}+S^{9}$	2
		$S^{6}c+S^{7}b$	1			$S^{8}a+S^{9}b$	1
		S^{7}	2			S^{9}	2
		$S^{7}+S^{8}b$	1			$S^{9}b$	1
		$S^{7}+S^{8}+S^{9}$	1			$S^{9}+S^{10}$	3
		$S^{7}a+S^{9}b$	1			S^{10}	4
		$S^{7}aii+S^{8}b$	1			$S^{10}b+c$	2
		$S^{7}b+S^{10}$	1				
		S^{8}	3				
		$S^{8}b$	1				
		$S^{8}+S^{9}$	4				
		$S^{8}+S^{9}a$	2				
		$S^{8}+S^{9}+S^{10}$	1				
		S^{10}	1				
		$S^{10}b+c$	1				

Data are presented as resected segments, and number of patients.
